# Preparation and characterization of N-doped ZnO and N-doped TiO_2_ beads for photocatalytic degradation of phenol and ammonia

**DOI:** 10.1007/s11356-022-19421-6

**Published:** 2022-03-27

**Authors:** Hagar Mandor, Nevine K. Amin, Ola Abdelwahab, El-Sayed Z. El-Ashtoukhy

**Affiliations:** 1 Chemical Engineering Department, Faculty of Engineering, Alexandria University, Alexandria, Egypt; 2 National Institute of Oceanography and Fisheries, NIOF, Alexandria, Egypt

**Keywords:** Photocatalytic degradation, N-doped ZnO, N-doped TiO_2_, Phenol, Ammonia

## Abstract

N-doped ZnO beads (NZB) and N-doped TiO_2_ beads (NTB) were synthesized via a modified sol–gel technique utilizing chitosan (CS)/polyvinyl alcohol (PVA) hydrogel beads as basic support for photocatalyst. Urea was used as a source of nitrogen in the preparation of N-doped ZnO beads, while ammonium acetate, CH_3_COONH_4_, was used as a nitrogen source in the production of N-doped TiO_2_ beads. The characteristics of synthesized beads were identified by scanning electron microscope (SEM), X-ray photoelectron spectroscopy analysis (XPS), X-ray diffraction (XRD), N_2_ adsorption–desorption isotherms, BET surface area, Fourier transform infrared (FT-IR) measurements, and diffuse reflectance spectroscopy (DRS) studies. The use of the nitrogen doping method for photocatalyst was performed to adjust the bandgap and electrical properties of ZnO and TiO_2_ by establishing acceptor defects. NZB and NTB with the intrinsic donor defect of oxygen vacancy and the nitrogen-to-oxygen acceptor defect could be activated by a less-energy UV consumption for efficient pollutant degradation. The results indicated that the as-synthesized NZB achieved much higher degradation activity than NTB, commercial ZnO, and TiO_2_ in the decomposition of a binary mixture composed of ammonia and phenol under UV light irradiation.

## Introduction

The world is nowadays moving towards a stage where water is going to be the main treasure of humankind because our life may cease to exist without water. The United Nations had pronounced the theme of World Water Day 2017 to be “wastewater” which reveals that the water the world needs should be sustainable and wastewater should be treated to get rid of the toxic pollutants before disposal or reuse (Debnath and Gupta 2018).

Phenol with a high concentration is produced in diverse wastewaters as petroleum, petrochemicals, and chemical industries. Sometimes ammonia nitrogen, heavy metals, etc. are discharged along with phenol in wastewaters as synthetic fuel processing and coal gasification wastewater (Sahariah and Chakraborty 2011). Phenol and its derivatives are usually supposed as probably the foremost significant organic pollutants discarded into the environment, recognized by the US Protection Agency as one of the most toxic pollutants (Boukhatem et al. 2017), resulting in significant harmful effects to human health and the environment (Valdés et al. 2015) and considered as carcinogenic compounds (Chen et al. 2016). Phenols are destructive to living organisms even at low concentrations (Grabowska et al. 2012).

Ammonia is a valuable and significant substance in the synthetic and industrial plants; however, at excessive concentration, it could accelerate the eutrophication in natural water as this phenomenon causes an improvement for algal growth which raises the toxicity in aquatic life (Mohammadi et al. 2016). Furthermore, in human consumptions, the high concentration of ammonia in drinking water decreases the capability of chlorine disinfection. Accordingly, to protect the world from ammonia harmful effects, the World Health Organization authorizes that the utmost concentration of ammonia in drinking water is 1.24 mg.L^−1^ (Mohammadi et al. 2016). Both phenolic compounds and ammonia nitrogen are the common toxic pollutants of the coal industry, petroleum refining plants, etc.

Many studies have been examined for the treatment of wastewater including phenol and ammonia nitrogen such as sequential anaerobic–anoxic–aerobic (Sahariah and Chakraborty 2011), ion exchange (Ricardo et al.^2012^), breakpoint chlorination (Eilbeck 1984), air stripping (Degermenci et al.^2012^), adsorption (Huo et al. 2012), anoxic–aerobic system with suspended growth reactors (Sahariah and Chakraborty 2011), electrochemical oxidation (Boukhatem et al. 2017), fixed-bed biofilm reactors, fluidized bed reactors, and membrane-based reactors (Sahariah and Chakraborty 2011). These processes produce wastes, for which additional treatment and managing demand further steps and expenses (Boukhatem et al. 2017; Mohammadi et al. 2016).

Due to these reasons, advanced oxidation processes (AOPs) have been suggested to get rid of ammonia nitrogen and organic pollutants, particularly those with low biodegradability (Peng et al. 2018; Asghar et al. 2015). AOPs have many merits such as the rate of photodegradation is rapid, mineralization of organic compounds, operating at ambient temperature and pressure, and reducing organic compounds toxicity (Mandal et al. 2010). The photodegradation process is considered as a sort of advanced oxidation process and has a significant degradation rate by developing hydroxyl radicals (OH•). This process has been investigated by numerous researchers as this process is compatible with the environment, and the cost of this process is low (Khan et al. 2017, 2018; Karthik et al. 2018; Malakootian et al. 2019).

Various semiconductors such as TiO_2_, CdS, ZnO, Fe_2_O_3_, and ZnS have been utilized as photocatalysts in the remediation of water and wastewater. There are some advantages and disadvantages for these semiconductors in UV irradiation (Mohammadi et al. 2016). It has been verified that TiO_2_ and ZnO are the most suitable photocatalysts for environmental applications because of their high effectiveness, chemical strength, non-toxicity, low cost, and reusability (Mahmodi et al. 2013; Merajin et al. 2013; Yazdanpour and Sharifnia 2013). However, TiO_2_ and ZnO have a significant recombination rate of photogenerated electrons and holes, causing a reduction in photodegradation process efficiency (Mohammadi et al. 2016; Shavisi et al. 2016). Also, one of the considerable constraints in the utilization of the photodegradation process in wastewater remediation is the accumulation during utility and the separation of powder from solution after reaction (Mohammadi et al. 2016; Jiang et al. 2014).

The use of three-dimensional, water floating, photocatalysts for water purification could be a good alternative since such materials can move on the top water, improving interactions with the pollutants in water. Also, they can receive a maximum irradiation, resulting in high photocatalytic efficiency even for the purification of highly concentrated pollutant wastewaters. Additionally, the recovery of floating photocatalysts and their recycling can be carried out easily (Djellabi et al. 2021). To improve the photocatalytic degradation activity, numerous techniques as metal or non-metal doping, dye sensitization, development of microspheres, and coupling semiconductors together have been proposed (Sahu et al. 2009; Loryuenyong et al. 2012; Choi et al. 2008).

Chitosan (CS) is the deacetylated derivative of chitin, the most common natural polymer discovered on earth after cellulose, produced from crustaceans like shrimps, squids, and crabs (Farzana and Meenakshi 2015). Chitosan is the main amino polysaccharide dispersed in considerable quantities in the earth (Muzzarelli 1996) and is utilized in numerous industries involving wastewater treatment, due to its biocompatibility, bi-functionality, and biodegradability (Cho et al. 1999; Crini 2006).

Currently, CS/polyvinyl alcohol (PVA) hydrogel beads are considered as ideal adsorbents for the treatment of aqueous solutions containing organic dyes and metal ions due to intermolecular interaction and creation of hydrogen bonds between CS and polyvinyl alcohol. Also, CS/PVA composite has proper mechanical property, special three-dimensional structure, appropriate film- and particle-forming property, and adjustable pore size. Due to these merits, CS/PVA hydrogel beads could be utilized such as an alternative template to form mesoporous materials (Jeun et al. 2009; Yang et al. 2004; Fajardo et al. 2012; Li et al. 2011).

Up to this time, even though there are considerable photocatalysts of metal-doped TiO_2_ and metal-doped ZnO, there are rare studies on non-metal-doped (Dindar and Guler 2018). It is recognized that boron (B) and nitrogen (N) are considered to replace atoms of oxygen.

Nitrogen doping has owned a major concern because the nitrogen and oxygen ionic radius is similar, the energy states of N 2p and O 2p also are similar, and highly soluble and formation energy is low (Dindar and Guler 2018). Accordingly, nitrogen in a low concentration (about 2–5% wt) was used to avoid the decreasing of bandgap energy for both two catalysts TiO_2_ and ZnO.

In consideration of the above aspects, this work provides a new technique for the development of photocatalytic beads. N-doped ZnO beads (NZB) and N-doped TiO_2_ beads (NTB) were formed by using CS/PVA hydrogel beads as a template to enhance photocatalytic activity. The beads were characterized by using SEM, EDAX, XPS, FT-IR, XRD, BET, and DRS. The photocatalytic activity of these beads was evaluated for simultaneous removal of a binary mixture which contains phenol and ammonia from aqueous solution. Furthermore, the role of nitrogen as a dopant and the mechanism of this method for the enhanced photodegradation activity were revealed.

## Materials and methods

### Materials

Zinc nitrate, chitosan, acetic acid, polyvinyl alcohol (98% hydrolyzed)), ammonia, sodium hydroxide, urea, zinc oxide, ammonium acetate CH_3_COONH_4_, titanium(IV) isopropoxide (TIP) (Sigma-Aldrich), 2-methoxyethanol, hydrochloric acid, titanium dioxide, deionized water, ammonium chloride, and phenol were used without any modification in experiments.

### Preparation of photocatalyst materials

#### N-doped ZnO bead photocatalyst

**Fig. 1:**
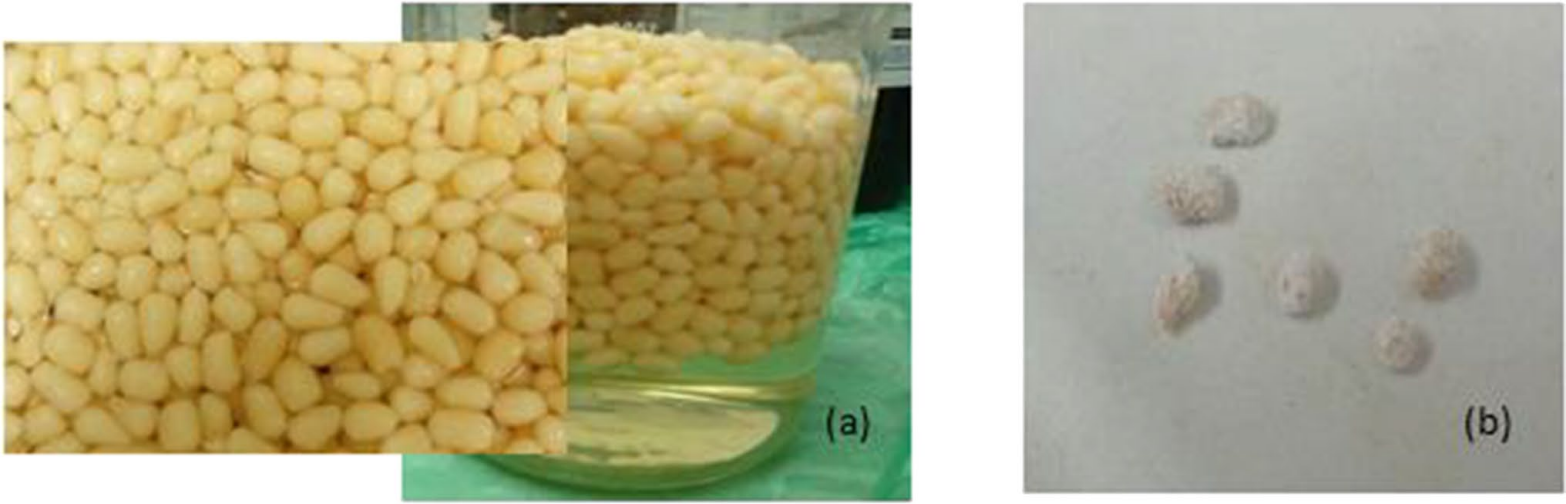
Composite NZB produced (**a**) in deionized water, and (**b**) after drying

N-doped ZnO beads were prepared by a modified sol–gel method using CS/PVA hydrogel beads as a template (Yuvaraja et al. 2017). Hydrogel beads were prepared by the following: dissolving 4 g of CS into 100 mL of 5% (v/v) aqueous acetic acid to form a CS solution. Four g of PVA was liquefied in 100 mL of deionized water to obtain PVA aqueous solution with stirring at 70 ± 1 °C. Then, the PVA solution was mixed homogeneously with CS solution with vigorous stirring for 3 h to obtain a composite gel-forming mixture (Yuvaraja et al. 2017). The resulting mixture was dropped into ammonia solution forming hydrogel beads, and then beads were soaked in sodium hydroxide bath (500 mL, 0.5 M) for 24 h for complete solidifying, and then washed with deionized water (Jiang et al. 2014). The resulted beads were soaked in the N-doped zinc solution for 48 h. The N-doped zinc solution was prepared as follows: 18 g of zinc nitrate was dissolved in 20 mL of NaOH (36%), and then the solution was diluted with 300 mL acetic acid and 100 mL deionized water (solution A). Five g of ZnO was mixed with 6 g of urea as a source of nitrogen and then added 60 mL of ethanol while stirring until complete evaporation of the solvent (solution B). The two solutions were mixed and then stirred for 3 h before use. After 48 h, the beads were filtered and kept in deionized water for 24 h to get rid of excess zinc solution. NZB beads were dried at 60 °C for 48 h as shown in Fig. 1.

#### *N-doped TiO*_*2*_* bead photocatalyst*

The composite gel-forming mixture was prepared as described above. The TiO_2_ nitrogen doping solution was formed by mixing ammonium acetate as a nitrogen source at 5 wt % with 3 ml of 2-methoxyethanol (Suphankij et al. 2013). Then, 8 ml titanium (IV) isopropoxide (TiP), 2 g of TiO_2_, and 9 ml of HCl were added to the nitrogen-doped TiO_2_ solution and stirred for 15 min. The produced nitrogen-doped solution was added to the composite gel-forming mixture with 20 ml of ethylene glycol. The resulting solution was vigorously stirred for 3 h. Afterward, the mixture was dropped into ammonia solution forming TiO_2_ beads.

**Fig. 2:**
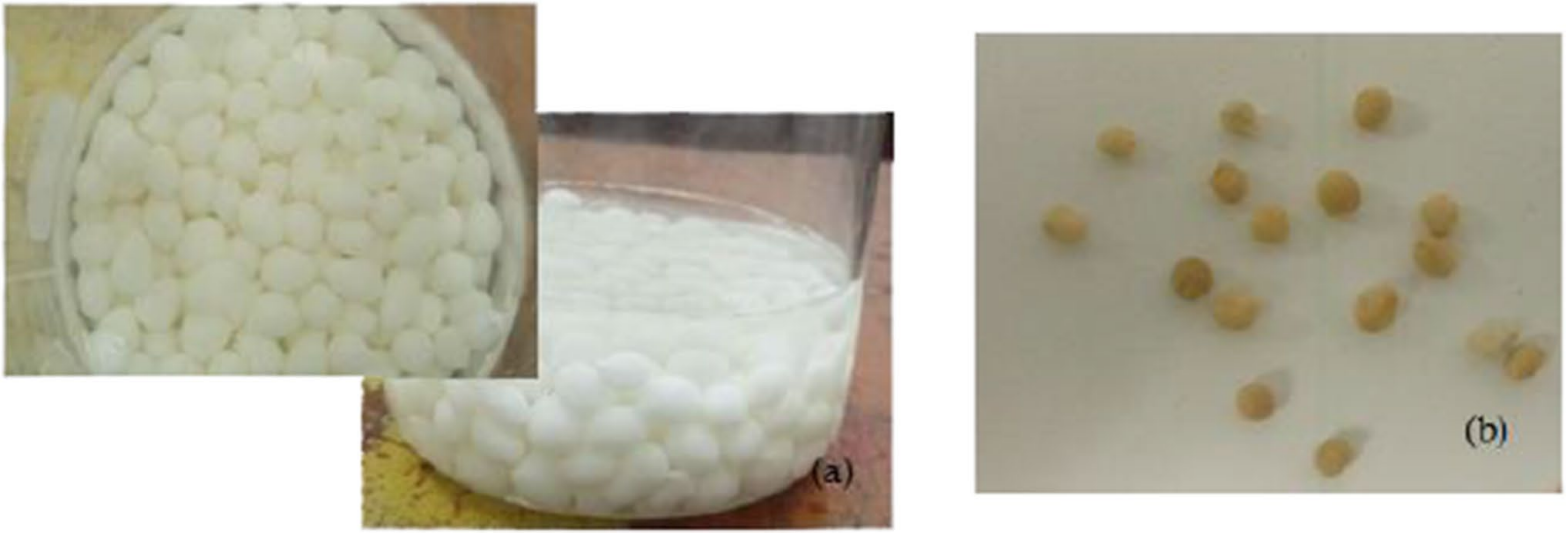
Composite NTB produced (**a**) in ammonia, and (**b**) after calcination

The beads were soaked in ammonia solution to complete the solidification for 1 h and then washed with double distilled water and kept in deionized water for a day. N-doped TiO_2_ beads were dried at 60 °C for 12 h. Finally, the beads were calcined at 200 °C for 15 min. The produced beads are illustrated in Fig. 2.

### Characterization of photocatalysts

The composition of elements and surface configuration of samples were studied by utilizing a JSM-IT200 In Touch Scope with a fully integrated EDS which includes “live EDS analysis” (JSM-IT200, JEOL, Akishima, Tokyo).

The photocatalyst crystal structures were indicated by X-ray diffraction (XRD) analysis (Bruker MeasSrv (D2-205,530)/D2-205,530) with CuKa1 radiation (wavelength = 1.54060 A°) at 30 kV voltage and 10 mA current. The wide-angle diffraction pattern was taken over a 2θ angle range extending from 20° to 80°.

FT-IR spectra of photocatalysts ZnO, TiO_2_, NZB, and NTB were scanned from 4000 cm^−1^ to 500 cm^−1^ on a Bruker Vector 22 (AVATAR 360, Nicolet, Madison, USA) with KBr powder (sample/KBr = 1/200).

Nitrogen adsorption/desorption isotherms were determined by a Belsorp Mini II (Japan) at 77 K. BET and BJH isotherm models are used to determine specific surface area and physical characteristics of pores.

X-ray photoelectron spectroscopy (XPS) was investigated by K-ALPHA (Thermo Fisher Scientific, USA) with monochromatic X-ray Al K-alpha radiation − 10 to 1350 eV at pressure 10^−9^ mbar with full-spectrum pass energy 200 eV and narrow-spectrum 50 eV.

To investigate the optical characteristics, the diffuse absorbance spectra of samples were measured by JASCO V-570 UV–vis absorption spectrophotometer in the range from 250 and 850 nm.

### Evaluation of photocatalytic degradation efficiency

Photodegradation efficiency was measured by the degradation of ammonia and phenol. A 15 W UV lamp (with a wavelength of 365 nm, Sylvania F15WT) was utilized for UV illumination. The space between lamp and solution surface was 15 cm. Testing solution consists of a mixture of 300 mg/L ammonia and 100 mg/L of phenol. One thousand mL of the solution was added with 0.5 g/L of NZB or NTB. Testing solution and catalyst were loaded in a 1.5 L quartz reactor. The reactor was located on a magnetic stirrer and was covered with aluminum foil, to improve the radiation flux to the reactor. Before irradiation, the reactants were agitated for 30 min in the dark to set up the adsorption–desorption equilibrium between the solution and the photocatalyst. During the irradiation, samples were drawn from the solution at time intervals (5, 10, 15, 30, 60, 120, 150, 180 min) and filtered. The solution was analyzed for the concentration of pollutants. The photodegradation percent of each product was calculated.

The concentration of phenol solution is determined using Standard Method 5530, and the concentration of ammonia nitrogen was measured by using Standard Method 4500.

## Results and discussion

### Catalyst characterization

#### Scanning electron microscope (SEM)

**Fig. 3:**
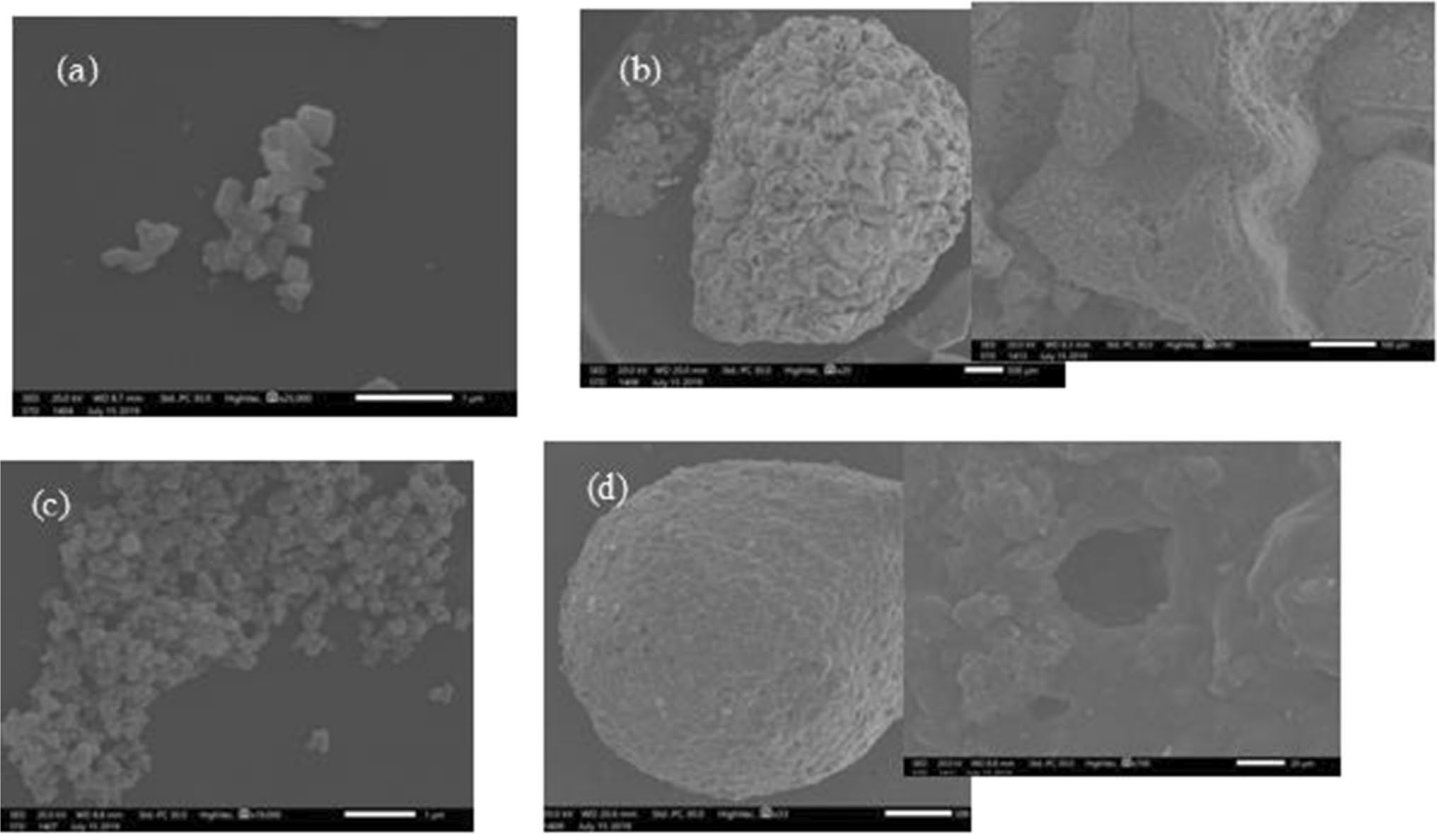
SEM images of a ZnO, b NZB, c TiO2, and d NTB

SEM is one of the most helpful analyses for investigating the surface morphology of the synthesized catalysts (Yuvaraja et al. 2017). The surface configuration of ZnO, NZB, TiO_2_, and NTB are shown in Fig. 3a–d.

Figure 3a shows that the surface of ZnO powder exhibited a nanoflake-like morphology; most of the ZnO sample appears as irregular rod-like or cubic-like particles. The surface of NZB is roughness with a pore structure. At 100 and 500 µm magnification, the NZB is arranged over one another in a flower shape. Also, NZB presented as irregular sphere-like or shutter-like particles with particle size 3.5–4.57 mm (Fig. 3b). Similar morphologies were reported by Farzana and Meenakshi (2015) and Wu et al. (2014).

The mesoporous TiO_2_ powder consisted of irregular particles that are shaped in clusters or clumps, as shown in Fig. 3c. The NTB catalyst had a well-defined spherical structure, and spheres had an average particle size of (2.75–3.2 mm), as shown in Fig. 3d. At high magnification images, the individual spheres or clumps have a mesoporous structure. SEM indicated that pore channels appear as wormholes. Till now, the mechanism of the porosity created on the film surface after adding TiO_2_ into CS/PVA composite is not properly revealed. Some researches clarified this mechanism based on a hypothesis that titanium dioxide molecules probably bind to CS particles with centrifugal forces which are exerted through preparation and calcination processes (Jiang et al. 2014). Also, similar morphologies have been reported by Jiang et al. (2014) and Kim et al. (2013).

#### EDX analysis

Chemical composition and purity of samples were tested by EDX studies for ZnO, NZB, TiO_2_, and NTB. For both ZnO and TiO_2_, the presence of Zn, Ti, and O are the only elements detected as shown in Table 1. EDX analysis of NZB and NTB shows the existence of C and N elements along with Zn, Ti, and O in catalysts NZB and NTB as shown in Table 1. This proves that CS was mixed well with ZnO to produce NZB and mixed with TiO_2_ for NTB. Also, the loading of nitrogen was confirmed by EDX for both NZB and NTB.

**Table 1:** EDX analysis for catalysts

Catalysts	Elements (Weight %)							
	O K	Zn K	Ti K	C K	N K	Na K	Cl K	Cu K
NZB	36.28 ± 0.32	42.01 ± 0.60	-	12.34 ± 0.19	2.48 ± 0.13	6.90 ± 0.25	-	-
NTB	43.10 ± 0.83	-	9.62 ± 0.25	37.25 ± 0.38	5.12 ± 0.51	0.77 ± 0.08	4.14 ± 0.13	-
ZnO	23.53 ± 0.24	76.47 ± 0.79	-	-	-	-	-	-
TiO_2_	46.08 ± 0.59	0.89 ± 0.10	30.77 ± 0.27	21.36 ± 0.16	-	-	-	0.90 ± 0.09

#### X-ray photoelectron spectroscopy analysis

**Fig. 4:**
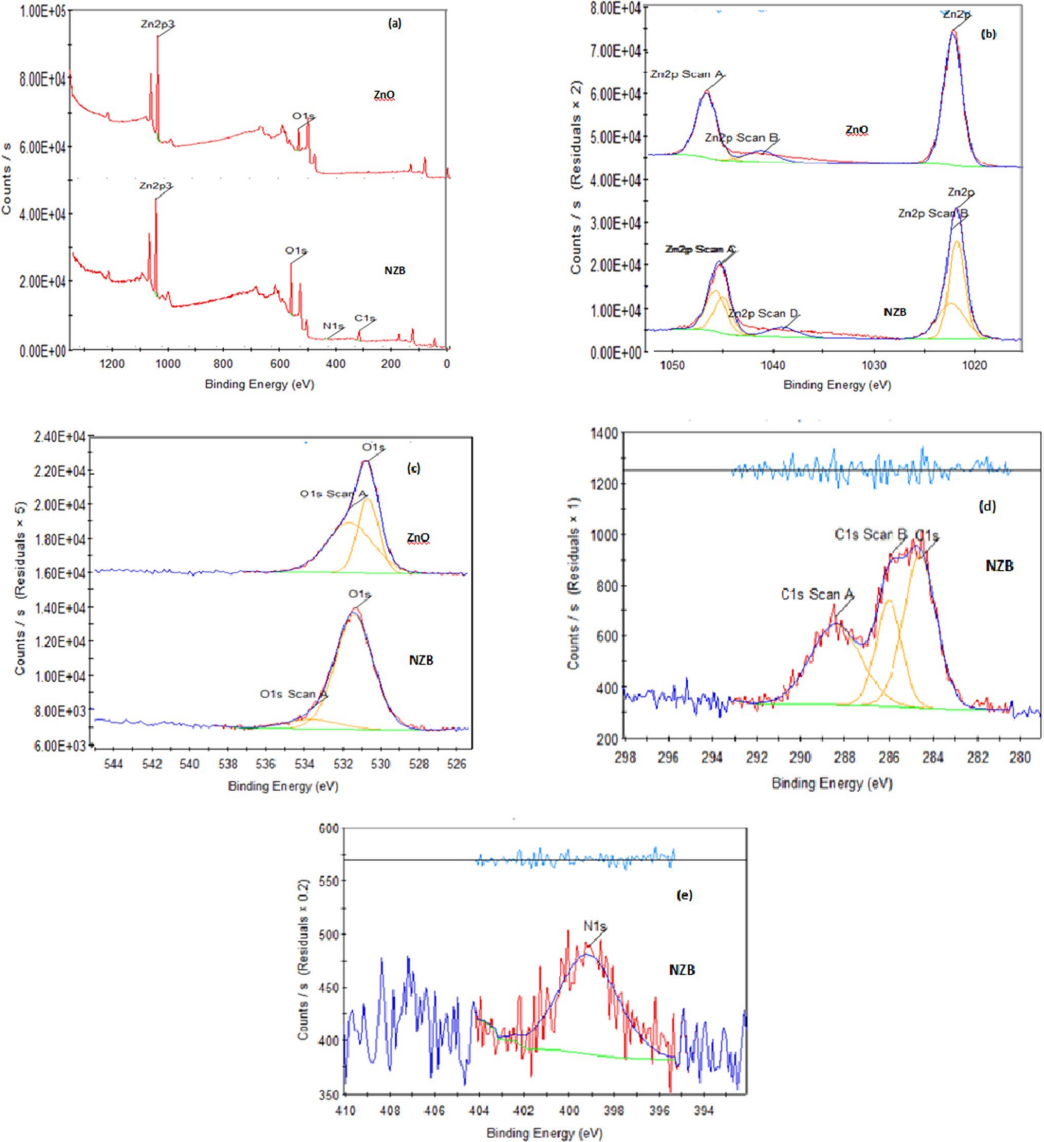
XPS scan of NZB and ZnO. **a** Survey scan, **b** Zn scan, **c** O scan, **d** C scan, **e** N scan

XPS survey was utilized to define surface components and binding energies of each element probably present in catalysts. The XPS results of NZB and ZnO are presented in Fig. 4a–e. The full XPS scan of the NZB and ZnO (Fig. 4a) revealed only peaks that were related to Zn, O, C, and N, while no peaks were indicated for the other elements. High-resolution XPS spectra of the Zn 2p lines are shown in Fig. 4b. There are two specific peaks of Zn 2p at 1048.38 (Zn 2p1/2) and 1022.28 eV (Zn 2p3/2) which refer to the oxidation state of Zn which was + 2 in the form of ZnO for all samples ( Wu 2014; Meenakshi et al. 2016; Singh et al. 2017; Chen et al. 2019).

The XPS spectra of O 1 s in Fig. 4c show that oxygen appears at binding energies, 530.4 and 532.2 eV for ZnO, while peaks appeared at 531.8 and 534.8 eV for NZB catalyst. Because oxygen atoms integrated with zinc atoms, there is a peak at 530.4 eV. The absorption peak at 532.2 eV assigns to adsorbed oxygen on the surface (Wu 2014; Meenakshi et al. 2016; Singh et al. 2017). Regarding NZB catalyst, peak at 531.8 eV is corresponding to bonds Zn–O–C and C–O–C (Chen et al. 2019) which appeared due to the presence of chitosan, and peak at 534.8 eV is assigned to bonds between oxygen and carbon in the form of C = O and due to presence of hydroxyl group on the surface of beads (Chen et al. 2019). Besides, as appeared in Fig. 4c, there is a shifting in binding energies for NZB, and this is referred to as the substitution of nitrogen atoms to oxygen atoms in ZnO lattice.

**Fig. 5:**
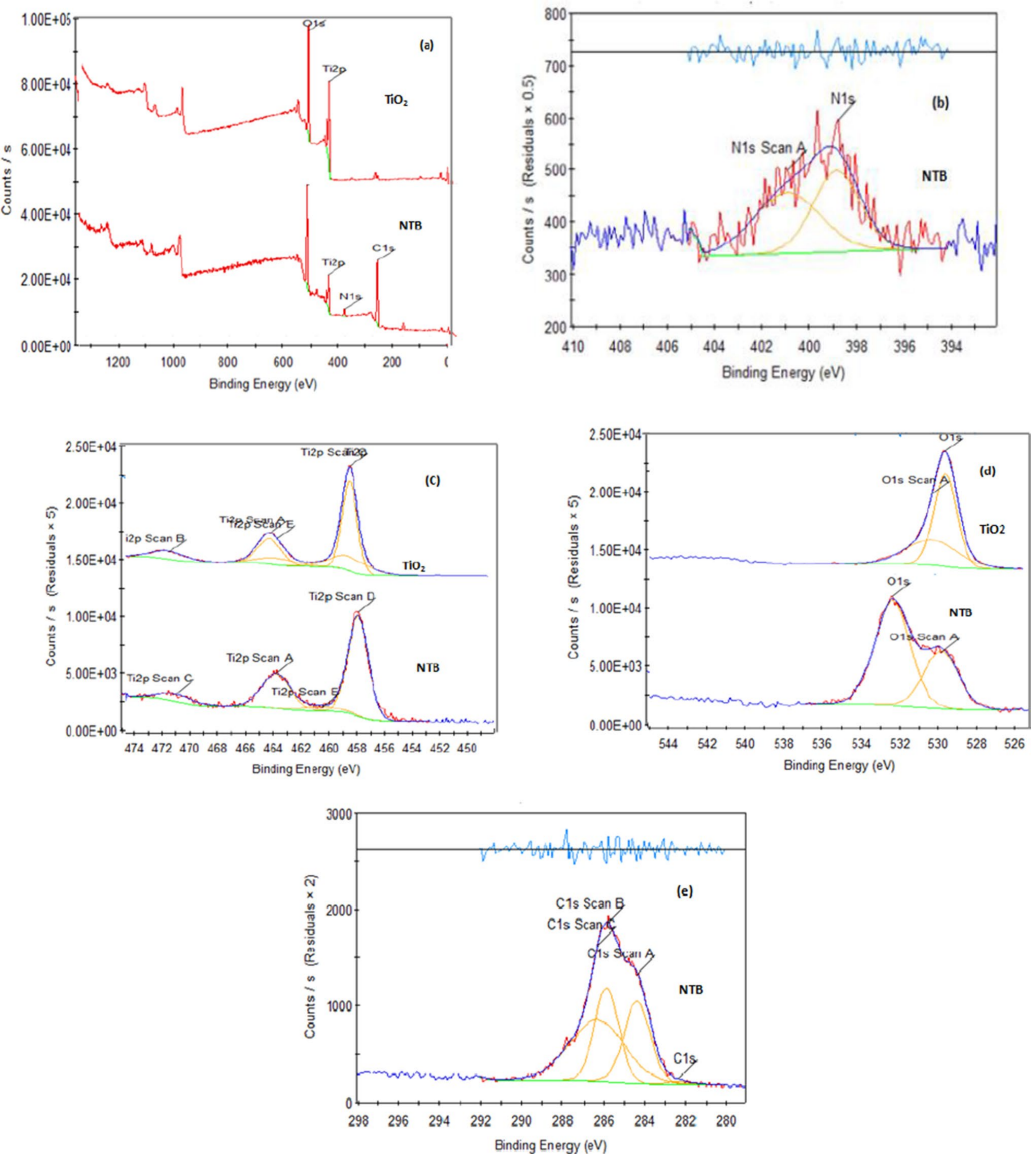
XPS scan of N–Ti beads and TiO_2_. **a** Survey scan, **b** N scan, **c** Ti scan, **d** O scan, **e** C scan

The XPS spectra of C 1 s can be decomposed into three peaks as shown in Fig. 4d. Peaks at 284.61 and 286 are assigned to sp2- and sp3-hybridized carbon atoms, respectively, which are due to its major configuration of carbon (Chen et al. 2019). It was noticed that the peak at 288.5 eV was weak and broadened peak which could be corresponding to bonds between carbons and oxygen as − C–OH, − C = O, and − C–O–Zn bonds (Meenakshi et al. 2016;Chen et al. 2019). Figure 4e shows that N1s peak appeared at 399.9 eV; this peak had a weaker signal as nitrogen was doped into ZnO lattice at a low concentration as illustrated in EDAX analysis; and this peak can be referred to N–H bond (Wu 2014). Figure 5a shows the XPS survey spectra of NTB and TiO_2_. There are four peaks with binding energies, 458.8, 531, 398.7, and 284.8 eV, which are assigned to Ti2p, O1s, N1s, and C1s, respectively, which confirmed the existence of Ti, O, N, and C elements in NTB.

The chemical states of N atoms in NTB were measured at N1s core levels as shown in Fig. 5b. There are two peaks at 398.7 eV and 400.2. The peak at 398.7 eV was assigned to bonds between nitrogen and titanium atoms (Ti–N bonds) in TiO_2_ lattice for NTB (Sun et al. 2008). However, in the case of Ti–N crystal, this peak appears at a binding energy of 396.9 eV (Sun et al. 2008). There is a difference of 1.8 eV which was assigned the binding energy of 1 s electron of N atom in O–Ti–N lattice. This difference might be clarified by the phenomenon that while nitrogen atoms superseded oxygen atoms in O–Ti–O lattice, there is a reduction in electron density around nitrogen compared to that in N–Ti–N lattice of Ti–N crystal, and this is because the oxygen atoms have higher electro-negativity than nitrogen atoms (Kim et al. 2013; Sun et al. 2008; Song et al. 2017; Cheng et al. 2012; Mohamed et al. 2015). The peak at 400.2 eV was corresponding to absorbed nitrogen molecules “γ-N2” which could form the following linkages: Ti–N–O or Ti–O–N (Kim et al. 2013; Sun et al. 2008).

High-resolution XPS spectra of Ti 2p spectra for NTB and TiO_2_ are shown in Fig. 5c. The peaks at 464.0 and 459 eV are assigned to Ti 2p1/2 and Ti 2p3/2, respectively, for the TiO_2_ sample, and this referred to that TiO_2_ existence in the form of Ti4 + ions (Kim et al. 2013). But in the case of nitrogen doping for NTB, the peaks occurred at 458.5 eV and 463.1 eV, respectively. The decrease in the binding energy of Ti 2p revealed that nitrogen was completely combined into the TiO_2_ structure (Kim et al. 2013; Song et al. 2017).

Figure 5d indicates the O 1 s spectrum for the samples. For the TiO_2_ sample, there are two peaks of the O 1 s region which appeared at 531 eV and 532.5 eV. The peak at binding energy 531 eV could be assigned to oxygen bonds in the TiO_2_ structure, whereas the peak at 532.5 eV might be attributed to the oxides and hydroxides of metal (Kim et al. 2013; Lee et al. 2016). Regarding the NTB sample, the peaks of the O 1 s spectra shifted significantly to low levels of binding energy which indicates the coexistence of nitrogen into the TiO_2_ lattice.

The C 1 s fine XPS spectrum of NTB is shown in Fig. 5e. Three peaks appeared at 284.8, 286.2, and 287.4 eV. The peak at 284.8 eV is corresponding to carbon bonds with carbon (C–C), while the peak at 286.2 eV is assigned to carbon bonds with oxygen and hydroxide group in the form of the following bonds: C–O and C–OH (Song et al. 2017; Lee et al. 2016). The final peak at 287.4 eV is referred to as bonds between carbon and oxygen in the form of C = O “COO” (Song et al. 2017; Lee et al. 2016).

#### X-ray diffraction

**Fig. 6:**
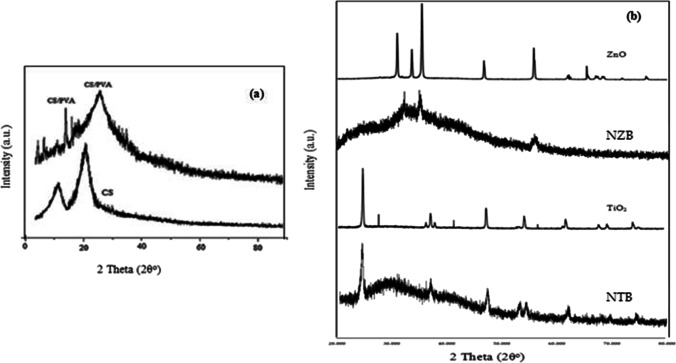
XRD patterns **a** CS and CS/PVA, **b** TiO_2_, NTB, ZnO, and NZB

XRD analysis was executed to evaluate the crystallinity and configuration of the catalysts. The patterns of samples (CS, CS/PVC, TiO_2_, ZnO, NZB, NTB) are illustrated in Fig. 6. Pure CS showed two peaks at angle 10° and 20°, while CS/PVA composite (Fig. 6a) exhibited two peaks one at 11.5° “low intensity” and 19.7° “high intensity” which are similar to the other researches (Yuvaraja et al. 2017). The presence of CS and PVA within the composition of both two catalysts NZB and NTB procured some changes in XRD for these two catalysts in comparison with pure ZnO and TiO_2_ (Fig. 6b). Furthermore, the sharp and narrow peaks revealed that the as-synthesized samples were crystalline, and no extra impurity peaks were observed which indicated the adequate purity of samples.

XRD pattern for ZnO reveals the significant peaks at (100), (002), (101), (102), (110), (103), (200), and (112) indicated adequate compatibility with wurtzite crystal structure which revealed the structure of crystals is hexagonal (Yuvaraja et al. 2017). The sharp diffraction patterns implies that zinc oxide particles are significant crystalline structure while for NZB by mixing CS/PVA with ZnO and nitrogen exhibit two strong peaks (100) and (110).

TiO_2_ and NTB catalysts displayed diffraction characteristic peaks observed at 2θ = 25.5°, 38.0°, 48.2°, 54.5°, 55.3°, 63.06°, 69.2°, 70.4°, and 75.2° which referred to (101), (104), (200), (105), (211), (204), (116), (220), and (215), respectively, and these assigned to anatase-phase levels (Suphankij et al.^2013^). The XRD patterns for NTB showed that nitrogen doping suppressed the conversion of anatase to brookite. Comparing XRD for NTB and TiO_2_ catalysts indicated that doping with nitrogen improved crystallinity resulting in an improvement of photocatalytic degradation activity (Cheng et al.^2012^).

Also, it was detected that the diffraction peak in the NTB was broadened due to the reduction in particle size including a destroying or collapsing in crystalline structure for catalyst (Cheng et al.^2012^). Furthermore, NTB diffraction peak intensity was lowered than TiO_2_ patterns, which may be assigned to the movement of nitrogen species into either the interstitial locations or the substitutional positions of TiO_2_ crystalline lattice (Cheng et al.^2012^).

The average sizes of the crystallites of TiO_2_, NTB, NZB, and ZnO were estimated using Scherrer equation (Farhadian et al.^2019^).

The average diameters of NTB and TiO_2_ at a diffraction peak 25.5° (2θ) were calculated as 17.47 and 10.06 nm, respectively. The average diameters of NZB and ZnO at a diffraction peak 33.5° (2θ) were calculated as 18.6 and 14.88 nm, respectively.

#### Specific surface area and porosity analysis

**Fig. 7:**
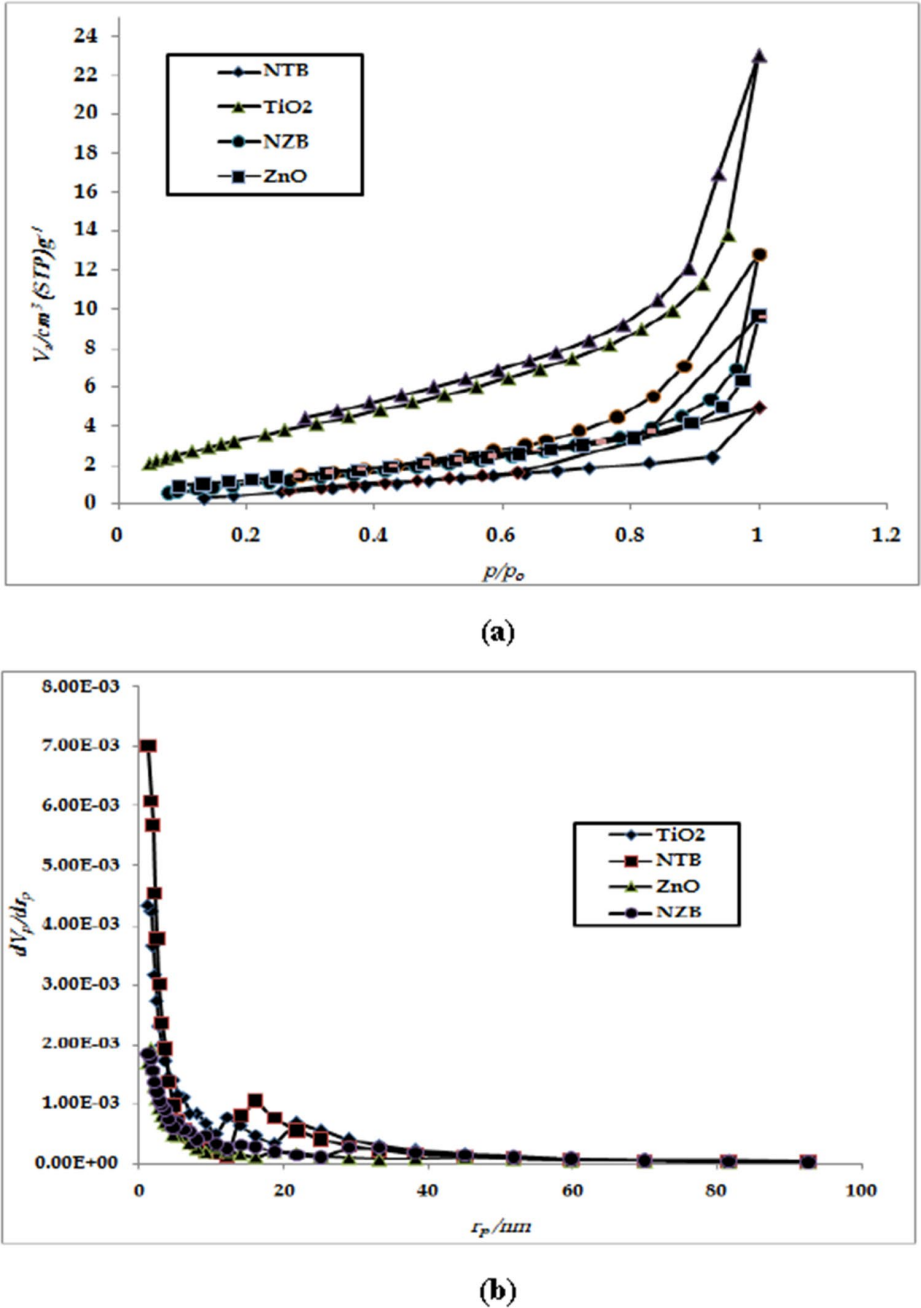
** a** N_2_-sorption isotherms (adsorption, desorption) and **b** pore size distribution (inset) curves for NZB, NTB, TiO_2_, and ZnO.

Barrett–Joyner–Halenda (BJH) pore size distribution plots and nitrogen adsorption–desorption isotherms of NZB, NTB, ZnO, and TiO_2_ are shown in Fig. 7a and b. It was detected that all catalysts have IV isotherms type and very narrow hysteresis loops at relative pressures approximately near to unity (Fig. 7a). That indicates the existence of a considerable well-developed mesoporous structure (size between 10 and 20 nm) (Jiang et al.^2014^; Kim et al.^2013^; Farhadian et al.^2019^; Zhou et al. 2018). Pore size distribution curves pointed out that the samples reveal maxima at 14.034 and 10.648 nm for NZB and ZnO, respectively, in addition to 16.138 and 12.203 nm for NTB and TiO_2_, respectively (Fig. 7b).

BET was used for the determination of surface area for NZB, NTB, ZnO, and TiO_2_. As shown in Table 2, the surface area of ZnO (36.428 m^2^g^−1^) was nearly two times higher than NZB photocatalyst. NZB surface area was smaller than pure ZnO, which could be explained by the fact that the small pores in Zn and then O sample shrunk through calcination (Kim et al.^2013^). The surface area of TiO_2_ (85.325 m^2^g^−1^) was nearly three times higher than that of the photocatalyst NTB which was calcined at 200 °C. As reported before, the calcination at higher temperatures may cause a breakdown in pore structure and enlargement of particle size (Jiang et al.^2014^).

**Table 2:** Physical parameters of NZB, NTB, ZnO, and TiO_2_

Material	Surface area (m^2^ g^−1^)	Pore volume (cm^3^ g^−1^)	Pore Diameter (nm)
NZB	16.757	1.1305	14.034
NTB	24.911	0.3666	16.138
ZnO	36.428	1.1392	10.648
TiO_2_	85.325	3.0948	12.203

The pore size of NZB and NTB is broader than ZnO and TiO_2_ due to the presence of CS/PVA composite which may be caused by an enlargement in the crystallinity of beads. Nevertheless, mesopores and macropores were developed through the accumulation of nanoparticles as the single of them is nonporous. These porous materials are considered valuable in photocatalysis because these materials can assist the atomic transportation of both reactants and products as reported before (Farhadian et al.^2019^).

## FT-IR analysis

**Fig. 8:**
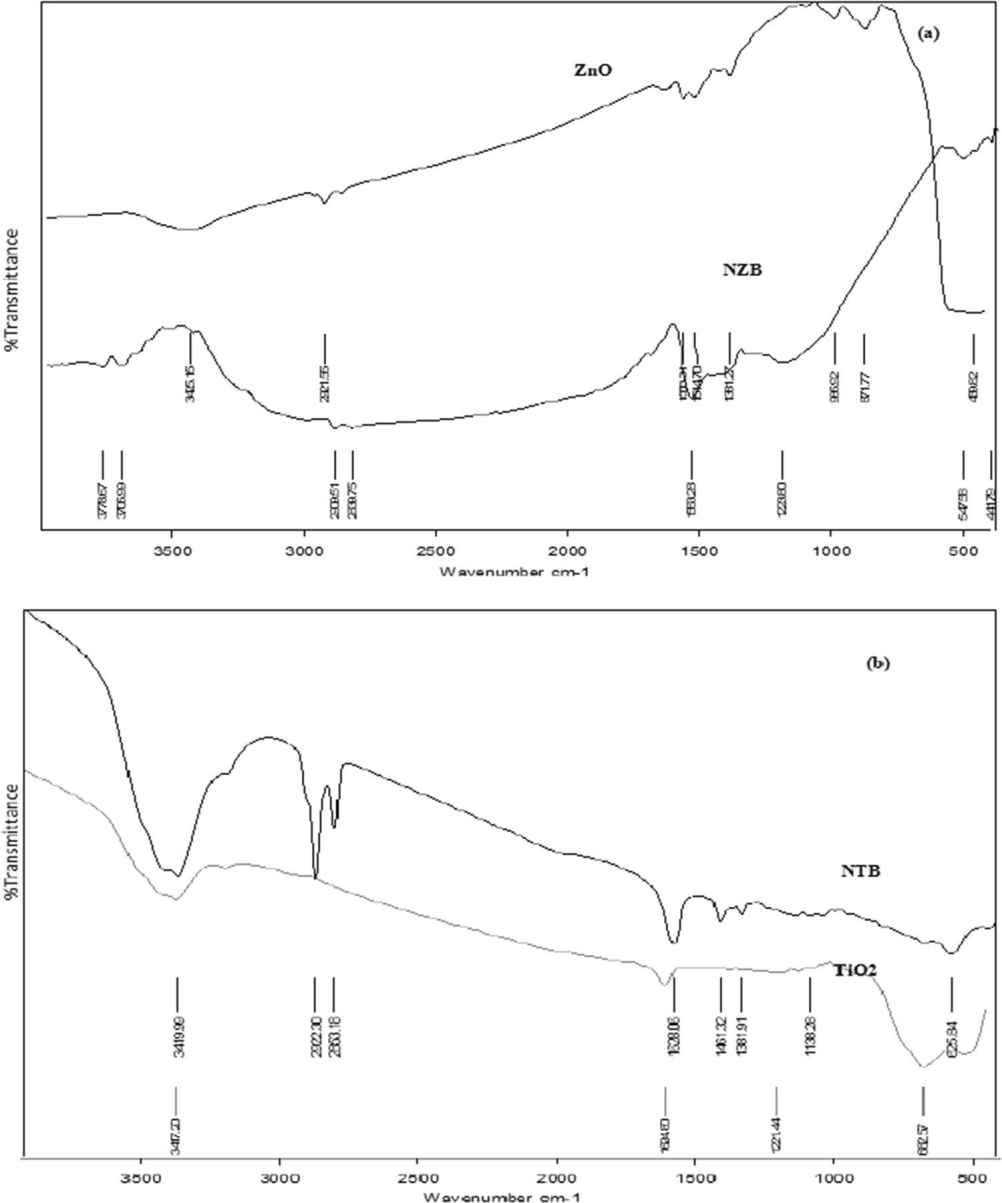
FT-IR spectra of **a** NZB and ZnO and **b** NTB and TiO_2_

The spectrum of FT-IR is exhibited in Fig. 8a–b for NZB, NTB, ZnO, and TiO_2_. The FT-IR spectra of four catalysts reveal a wide absorption band at 3400 cm^−1^ which referred to O–H stretching vibrations of adsorbed water on catalysts surface (Farzana and Meenakshi 2015). The absorption peaks at 2921 cm^−1^, which appears on spectra of NZB, NTB, and ZnO, are a result of asymmetric stretching of CH_3_ and CH_2_ groups (Farzana and Meenakshi 2015). For TiO_2_ and ZnO spectra, the peaks at 1624 cm^−1^ and 1550 cm^−1^ are corresponding to adsorbed H_2_O which was the only peak in the same region (Nolan et al.^2012^). A peak at 1380 cm^−1^ at NZB and ZnO spectra, peak at 1200 cm^−1^ at TiO_2_ spectra, and peak at 1085 cm^−1^ for NTB spectra revealed to C–O stretch (Jiang et al.^2014^). Both TiO_2_ and NTB catalysts show a strong peak around 600 cm^−1^ which is caused by Ti–O–Ti lattice vibrations (Jiang et al.^2014^). Concerning NZB and ZnO spectra, there are peaks at 871 cm^−1^, 620 cm^−1^, 478 cm^−1^, and 459 cm^−1^ which were referred to the Zn–O stretching (Yuvaraja et al.^2017^).

As a result of the modification in the structure of TiO_2_ and ZnO, there is an absorption peak at 2850 at the spectra of NZB and NTB which was ascribed to the C–H stretching. There is also a peak at 1620 cm^−1^ which is due to NH_2_ vibrations which revealed that nitrogen is chelated to the center of titanium and zinc, rising its coordination number to six and developing a chelated complex (Nolan et al.^2012^).

## The diffuse reflectance spectroscopy (DRS) studies

**Fig. 9:**
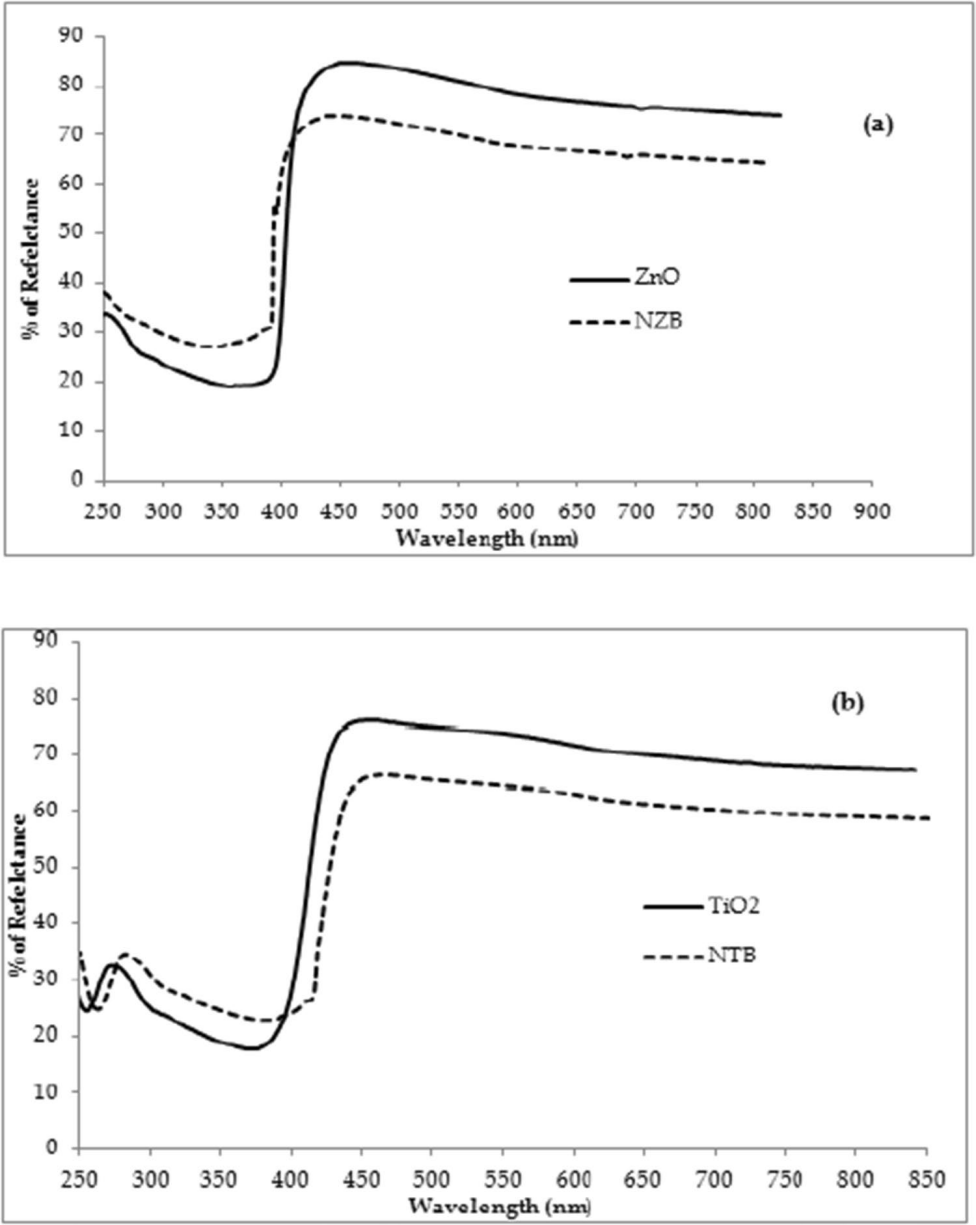
UV–vis absorption spectra: a ZnO and NZB photocatalysts and b TiO2 and NTB photocatalysts

To investigate the optical characteristics of the synthesized beads, their UV–vis absorption spectra were shown in Fig. 9a–b for NZB, NTB, ZnO, and TiO_2_. UV–vis spectroscopy is a powerful tool to inspect the light-absorbing nature of solid powders (Bechambi et al.^2016^).

The UV–vis absorption spectrum of undoped ZnO and NZB samples (Fig. 9a) shows an effective pattern in the region of UV light which are corresponding to the semiconductor optical characteristic (Bechambi et al.^2016^). Moreover, there is a slight red shift of the UV–vis absorption edge which was noticed for NZB compared to ZnO, but both spectra are near-ultraviolet (UV) region as reported before (Dindar and Guler 2018). The effective peak at 387.5 nm is a significant peak for the wurtzite hexagonal structure of pure ZnO.

The photocatalyst bandgap energy for NZB and ZnO is 3.16 eV and 3.2 eV, respectively (Mohammadi et al.^2016^; Farzana and Meenakshi 2015; Wu et al.^2014^; Singh et al.^2017^; Ramos-Corona et al.^2019^). In addition, there is a wide tail from approximately 400 nm to 800 nm exhibited in the spectrum of the NZB (Wu et al.^2014^). The shifting of absorption edge and the extra-wide tail which extended into the visible region for NZB compared to ZnO might be assigned to the following reasons: the enlargement in the crystal size and doping of nitrogen to ZnO lattice (Wu et al.^2014^).

The absorption spectrum of NTB catalyst was moved to lower energy region than pure TiO_2_ sample. Diffuse reflectance spectra of NTB as shown in Fig. 9b indicate the same light absorption in the near-ultraviolet zone as the pure TiO_2_. The absorption edge of TiO_2_ is at 380 nm. The bandgap energies for NTB and TiO_2_ are 3.19 eV and 3.26 eV, respectively (Mohammadi et al.^2016^; Song et al.^2017^; Jaiswal et al.^2015^; Pérez et al.^2015^). The reduction in bandgap energy was due to the effect of N-doping which assisted the electron capture and further enhanced the separation effectiveness of electron–hole pair recombination (Lee et al.^2016^). Additionally, the nitrogen doping in the TiO_2_ lattice modified the electronic band configuration of titania by merging the N 2p orbital with the O 2p orbital, hence causing the narrowing of bandgap of the material (Lee et al.^2016^; Jaiswal et al.^2015^; Pérez et al. 2015; Saien and Mesgari 2016).

### Evaluation of photocatalytic degradation efficiency of synthesized beads

**Fig. 10:**
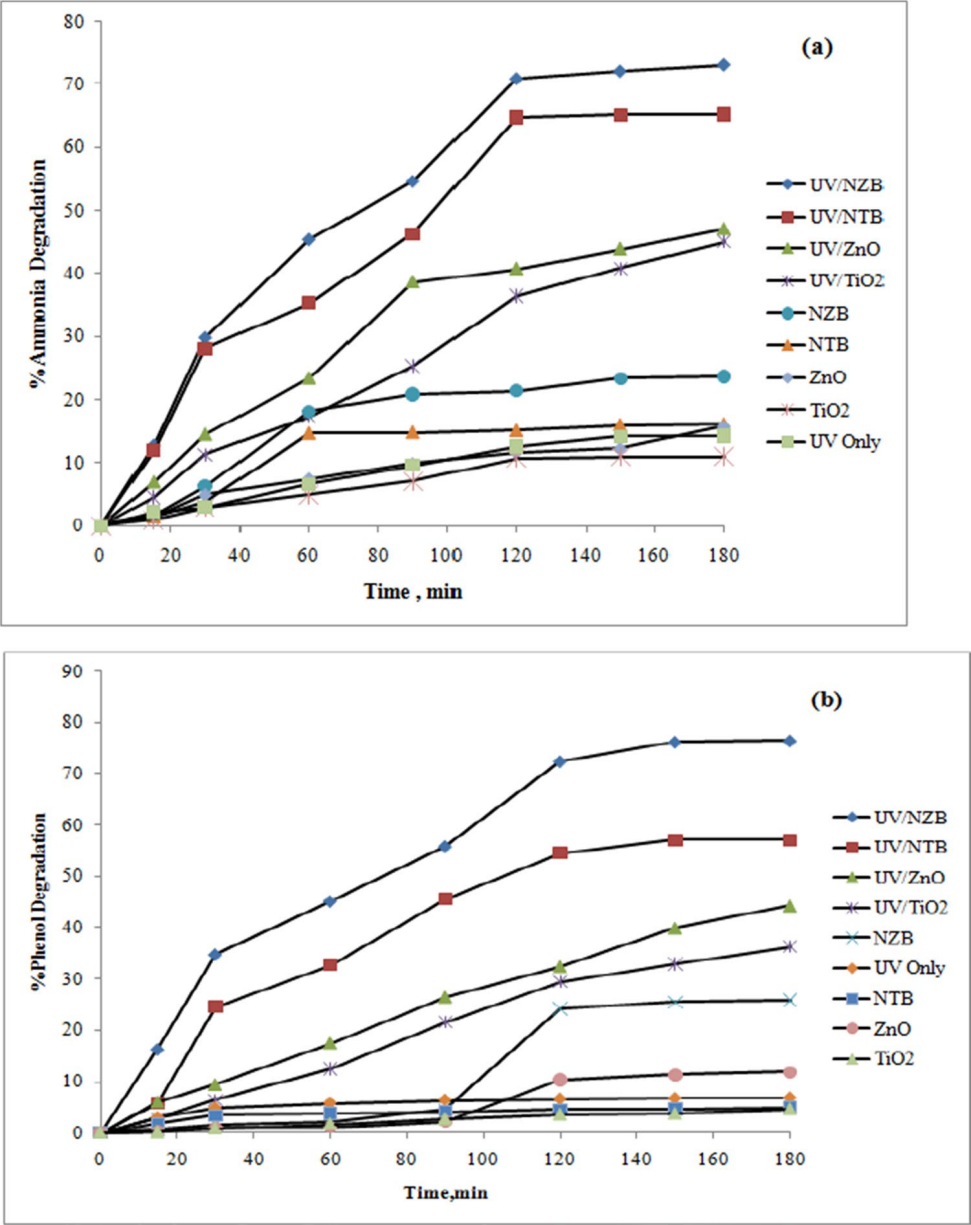
Photolysis, adsorption, and photocatalytic degradation of (**a**) ammonia, and (**b**) phenol under UV irradiations at different conditions

The photodegradation efficiency of synthesized samples was estimated by determining the degradation of ammonia and phenol in an aqueous solution. The efficiency was investigated with 100 ppm phenol initial concentration and 300 ppm ammonia initial concentration at neutral pH “6.2,” dose 0.5 g/L and 15 W UV lamp. The degradation efficiency of each sample (NZB, NTB, ZnO, and TiO_2_) and UV/catalyst in the removal of ammonia and phenol at the same conditions are shown in Fig. 10a–b.

It has been shown from Fig. 10a–b, the photodegradation of phenol and ammonia at direct photolysis alone leads to extremely low ammonia and phenol removal (14.22% ammonia and 6.27% phenol). In the existence of NZB, NTB, ZnO, and TiO_2_ without UV “in dark conditions,” the degradation of phenol and ammonia was very low as there is no adsorption of pollutants over the photocatalyst surface (for NZB, 23.70% ammonia and 25.83% phenol; for NTB, 16.15% ammonia and 4.941% phenol; for ZnO, 15.805% ammonia and 11.845% phenol; and for TiO_2_, 11.04% ammonia and 4.54% phenol). This is clarified by other researches that in case of a low photo-transformation quantum yield, i.e., the reactive excited states deactivate during non-photochemical processes (Boukhatem et al.^2017^). Additionally, it indicates the direct photolysis is not an effective process to eliminate ammonia and phenol in water which was reported by others (Boukhatem et al.^2017^; Shaveisi and Sharifnia 2018). As shown from Fig. 10a–b, the degradation of ammonia and phenol is greatly accelerated by the combination of UV with catalyst where the degradation efficiency of phenol and ammonia reached 47.032% and 45.021% ammonia and 44.269% and 36.282% phenol for both ZnO and TiO_2_, respectively. The degradation efficiency increased to 76.45% and 57.075% phenol and 73.061% and 69.278% ammonia for both NZB and NTB, respectively.

### Mechanism of photocatalytic degradation activity

**Fig. 11:**
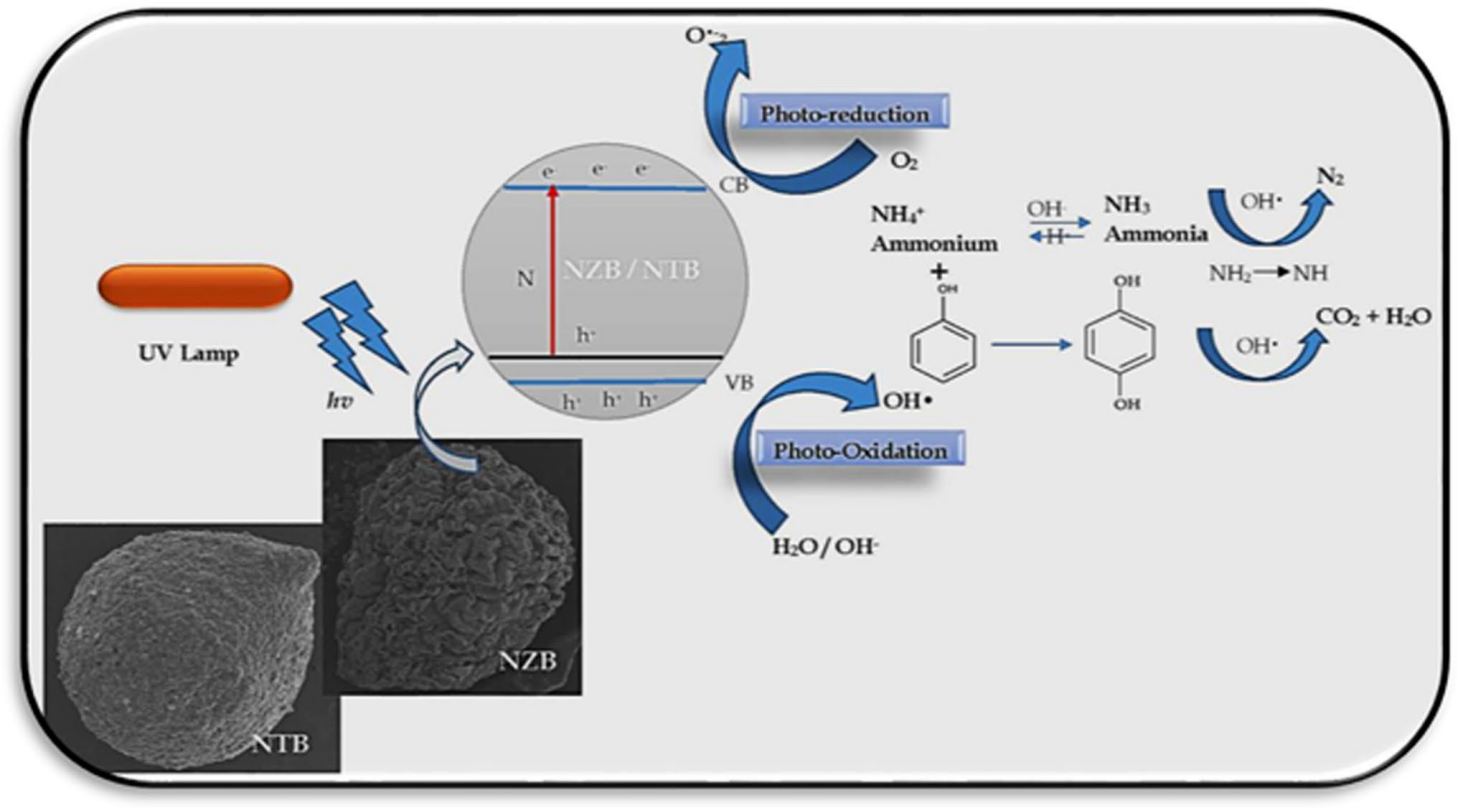
The mechanism of the photocatalytic degradation of ammonia and phenol by NZB and NTB beads

**Fig. 12:**
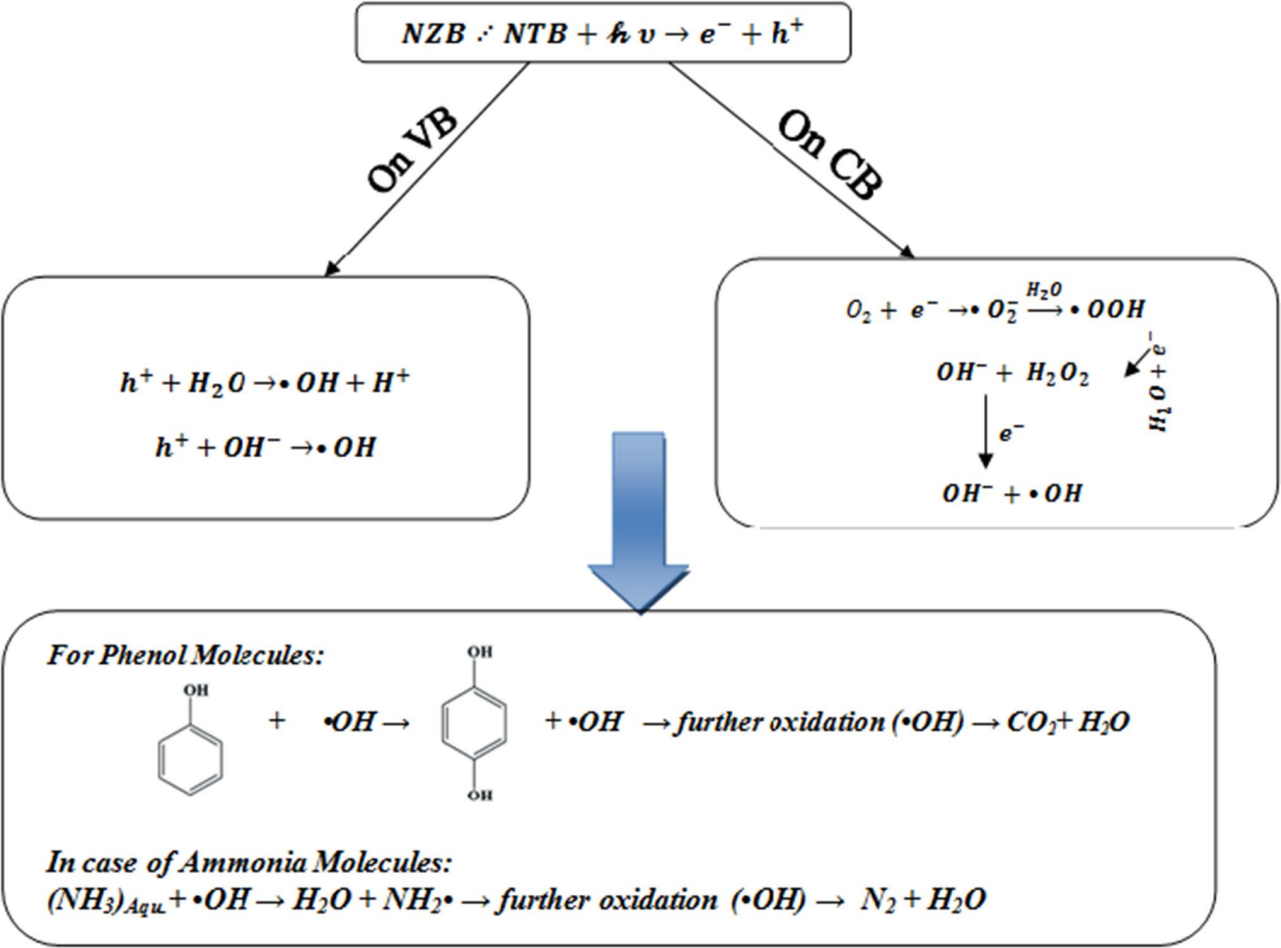
Mechanism of hydroxyl radical production at the surface of NZB/NTB

Under UV illumination, both two catalysts NZB and NTB can be excited to form e^−^–h^+^ pairs. Positive hole (h^+^) is developed in the valence band (VB), whereas electron is incited to the conduction band (CB). Nitrogen doping for photocatalyst will produce a new energy level formed in the bandgap of NZB and NTB by the diffusion of nitrogen particles in the photocatalyst structure as seen in Fig. 11. The electron can be activated from the defect state to the photocatalyst conduction band by a photon with energy equals *hv* (Grabowska et al.^2012^; Nolan et al.^2012^; Peng et al.^2019^). The presence of nitrogen can modify the band structure and control the recombination efficiency of the photogenerated electron–hole pairs during UV illumination. The reduction of the charge carrier’s recombination results in the improvement of photodegradation efficiency (Kim et al.^2013^; Nolan et al.^2012^). The production of hydroxyl radicals which are oxidizing agents as described by the flow chart in Fig. 12 can attack phenol and ammonia molecules that are presented near or at the surface of NZB and NTB which causes photocatalytic degradation of them according to the proposed mechanism.

Moreover, as we mentioned before, the use of photocatalyst nanoparticles is still facing limitations as the accumulation through utility and complexity in segregation and recovering them from treated effluent (Jiang et al.^2014^; Farhadian et al.^2019^). A substitutional favorable method to figure out these limitations is to use solid support which allows immobilizing these semiconductors on this solid support, and then it could be separated and reused after the reaction (Farhadian et al.^2019^). These supporting materials as chitosan should have effective characteristics such as easy recovery, controllable pore space and surface chemistry, and good mechanical strength for long-term use (Farhadian et al.^2019^). Accordingly, NZB and NTB proved they have excellent photocatalytic activity rather than the commercial ZnO and TiO_2_.

## Conclusions

A simple method was used to prepare NZB and NTB as an effective photocatalyst by using chitosan and PVA hydrogel beads as a template. The existence of nitrogen with a small amount in NZB and NTB photocatalysts was confirmed by using various techniques as EDX and XPS analysis. The sharp and narrow peaks exhibited for as-synthesized catalysts and the crystalline structure with no additional impurity peaks were observed which indicated the good purity of prepared beads by XRD analysis. The peak revealed by FT-IR at 2850 cm^−1^ could be attributed to C–H stretching due to using chitosan/polyvinyl alcohol for both NZB and NTB and peaks at 1620 cm^−1^ which resulted from NH_2_ vibrations revealed that nitrogen is chelated to the titanium and zinc metal center. The strong absorption peak by using the DRS technique at 387.5 nm for NZB and 388.7 nm for NTB confirmed a decrease in bandgap for the two catalysts due to the effect of N-doping which promoted the electron capture and facilitated the separation efficiency of the photo-induced electron–hole pairs. At UV illumination, the prepared catalysts presented better degradation efficiency for ammonia and phenol than commercial ZnO and TiO_2_ photocatalysts after 180 min of UV illumination time as the photodegradation percentage was 73.061% and 69.278% ammonia and 76.45% and 57.075% phenol for both NZB and NTB compared to 47.032% and 45.021% ammonia and 44.269% and 36.282% phenol for both ZnO and TiO_2_.

## Data Availability

All data generated or analyzed during this study are included in this published article.
